# Health Behaviours and Potentially Preventable Hospitalisation: A Prospective Study of Older Australian Adults

**DOI:** 10.1371/journal.pone.0093111

**Published:** 2014-04-01

**Authors:** Bich Tran, Michael O. Falster, Kirsty Douglas, Fiona Blyth, Louisa R. Jorm

**Affiliations:** 1 Centre for Health Research, School of Medicine, University of Western Sydney, Campbelltown, NSW, Australia; 2 Australian National University, Canberra, ACT, Australia; 3 The Sax Institute, Haymarket, NSW, Australia; 4 Concord Clinical School, University of Sydney, NSW, Australia; Newcastle University, United Kingdom

## Abstract

**Objective:**

Several studies have demonstrated the effects of health behaviours on risk of chronic diseases and mortality, but none have investigated their contribution to potentially preventable hospitalisation (PPH). We aimed to quantify the effects on risk of PPH of six health behaviours: smoking; alcohol consumption; physical activity; fruit and vegetables consumption; sitting time; and sleeping time.

**Design/Setting:**

Prospective observational study in New South Wales, Australia.

**Subjects:**

267,006 men and women aged 45 years and over.

**Outcome Measures:**

PPH admissions and mortality during follow-up according to individual positive health behaviours (non-smoking, <14 alcoholic drinks per week, ≥2.5 hours of physical activity per week, ≥2 servings of fruit and 5 servings of vegetables per day, <8 hours sitting and ≥7 hours sleeping per day) and the total number of these behaviours.

**Results:**

During an average of 3 years follow-up, 20971 (8%) participants had at least one PPH admission. After adjusting for potential confounders, participants who reported all six positive health behaviours at baseline had 46% lower risk of PPH admission (95% CI 0.48–0.61), compared to those who reported having only one of these behaviours. Based on these risk estimates, approximately 29% of PPH admissions in Australians aged 45 years and over were attributable to not adhering to the six health behaviours. Estimates were similar for acute, chronic and vaccine-preventable categories of PPH admissions.

**Conclusions:**

Individual and combined positive health behaviours were associated with lower risk of PPH admission. These findings suggest that there is a significant opportunity to reduce PPH by promoting healthy behaviours.

## Introduction

Potentially preventable hospitalisation (PPH, also termed avoidable or ambulatory care sensitive hospitalisation), defined on the basis of a set of diagnoses relating to chronic, acute and vaccine-preventable conditions [1], has been adopted by health systems internationally as an indicator of access to and quality of primary care [2]–[6]. However, there has been little attention to the mechanisms by which primary care may prevent these admissions, and the potential contributions of primary and secondary prevention [7].

Longitudinal studies from around the world have demonstrated that positive health behaviours, including non-smoking, low or moderate alcohol use, being physically active, and consumption of fruit and vegetables, are associated with reduced risk of chronic diseases [8], [9] and mortality [10]–[13]. Recently, evidence from observational studies has suggested that prolonged sitting time [14], [15] and short sleep duration [16], [17] increase the risk of mortality. However, there has been no comprehensive examination of the impact of health behaviours on the risk of PPH.

Much research on PPH has focused on socio-demographic or structural factors which moderate access to and quality of primary care, as well as the density of general practitioners, perceived availability of health services, presence of community health centres, and continuity in health service provision [18]. Previous studies have shown that demographic characteristics of participants such as older age, ethnic background, rural residential location and poor health status were associated with increased risk of PPH [19], [20].

Using individual-level data from a large prospective cohort study, linked to hospital morbidity and death data, we aimed to (i) quantify the individual and combined effects of six health behaviours (smoking, alcohol consumption, physical activity, fruit and vegetables consumption, sitting and sleeping time) on risk of PPH; and (ii) compare the magnitudes of these effects according to category of PPH admission.

## Methods

### Participants

This was part of the Assessing Preventable Hospitalisation InDicators (APHID) study [1]. APHID uses linked survey and administrative data for participants in the Sax Institute's 45 and Up Study, a prospective cohort of over 267000 men and women aged 45 years and above and resident in New South Wales (NSW), Australia [21]. Participants were randomly sampled from the database of the national health insurance scheme (Medicare Australia). Participants entered the study by completing a mailed self-administered questionnaire at baseline (between February 2006 and April 2009) and providing written consent for long-term follow-up and linkage of their health information to a range of routine health databases. People residing in non-urban areas and those aged 80 years and over were oversampled. The overall response rate for the 45 and Up Study is estimated to be 18% and the study included about 10% of the NSW population aged 45 and over.

### Data collection

Exposure and confounding variables used in this analysis were derived from self-reported data from the 45 and Up Study baseline questionnaire (available at https://www.saxinstitute.org.au/our-work/45-up-study/), apart from the measure of remoteness of residence, which was assigned according to the mean score of Accessibility Remoteness Index of Australia Plus (ARIA+) for the Postal Area of the participant's address [22]. History of chronic conditions was obtained from responses to the questions “Has a doctor ever told you have melanoma/prostate cancer/breast cancer/other cancer/heart disease/stroke/diabetes/asthma” and “In the last month, have you been treated for osteoarthritis?” Other variables were classified according to the groupings in **Table 1**, with an additional category for missing values.

**Table 1:** Characteristics of participants according to number of positive health behaviours.

	Number of positive health behaviours						
	0	1	2	3	4	5	6
N (% of total)	229 (0.1)	2816 (1.0)	16198 (6.1)	51457 (19.3)	91194 (34.2)	81963 (30.7)	23149 (8.7)
**Gender**							
Male	162 (70.7)	1672 (59.4)	9244 (57.1)	27875 (54.2)	45206 (49.6)	33561 (41.0)	6135 (26.5)
Female	67 (29.3)	1144 (40.6)	6954 (42.9)	23582 (45.8)	45988 (50.4)	48402 (59.0)	17014 (73.5)
**Age, years**							
45–50	50 (21.8)	437 (15.5)	2325 (14.3)	7353 (14.3)	12249 (13.4)	10173 (12.4)	2318 (10.0)
50–54	48 (21.0)	486 (17.3)	2770 (17.1)	8678 (16.9)	14928 (16.4)	12734 (15.5)	3336 (14.4)
55–59	52 (22.7)	475 (16.9)	2647 (16.3)	8695 (16.9)	15592 (17.1)	13998 (17.1)	4044 (17.5)
60–64	24 (10.5)	380 (13.5)	2154 (13.3)	7059 (13.7)	13384 (14.7)	13279 (16.2)	4089 (17.7)
65–69	16 (7.0)	259 (9.2)	1630 (10.1)	5504 (10.7)	11256 (12.3)	11400 (13.9)	3635 (15.7)
70–74	8 (3.5)	228 (8.1)	1274 (7.9)	4062 (7.9)	8324 (9.1)	7980 (9.7)	2501 (10.8)
75–79	10 (4.4)	202 (7.2)	1087 (6.7)	3418 (6.6)	6145 (6.7)	5510 (6.7)	1613 (7.0)
80+	21 (9.2)	349 (12.4)	2311 (14.3)	6688 (13.0)	9316 (10.2)	6889 (8.4)	1613 (7.0)
**Education**							
Did not complete high school	121 (52.8)	1282 (45.5)	6420 (39.6)	18214 (35.4)	29909 (32.8)	26212 (32.0)	7916 (34.2)
High school or equivalent	72 (31.5)	997 (35.4)	6224 (38.4)	20712 (40.2)	38085 (41.8)	35136 (42.9)	9694 (41.9)
University or higher	22 (9.6)	338 (12.0)	3005 (18.6)	11470 (22.3)	21815 (23.9)	19594 (23.9)	5302 (22.9)
Missing	14 (6.1)	199 (7.1)	549 (3.4)	1061 (2.1)	1385 (1.5)	1021 (1.2)	237 (1.0)
**Marital status**							
Single	45 (19.6)	337 (12.0)	1322 (8.1)	3447 (6.7)	5172 (5.7)	3946 (4.8)	915 (4.0)
Married or partnered	103 (45.0)	1564 (55.5)	10397 (64.2)	36161 (70.3)	67919 (74.5)	63544 (77.5)	18398 (79.5)
Widowed or separated	75 (32.8)	869 (30.9)	4336 (26.8)	11468 (22.3)	17527 (19.2)	14090 (17.2)	3754 (16.2)
Missing	6 (2.6)	46 (1.6)	143 (0.9)	381 (0.7)	576 (0.6)	383 (0.5)	82 (0.3)
**Household income**							
< 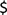 10,000	28 (12.2)	305 (10.8)	1322 (8.2)	3214 (6.3)	4895 (5.4)	4117 (5.0)	1054 (4.5)
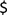 10,000– 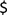 29,999	50 (21.8)	662 (23.5)	3718 (22.9)	11796 (22.9)	21123 (23.2)	19989 (24.4)	5853 (25.3)
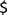 30,000– 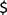 49,999	26 (11.4)	313 (11.1)	1905 (11.8)	6987 (13.6)	13931 (15.3)	13155 (16.0)	6064 (17.6)
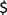 50,000– 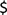 69,999	9 (3.9)	234 (8.3)	1462 (9.0)	5212 (10.1)	9612 (10.5)	8900 (10.9)	2433 (10.5)
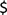 70,000 or more	34 (14.9)	487 (17.3)	3685 (22.7)	12910 (25.1)	22542 (24.7)	18466 (22.5)	4672 (20.2)
Prefer not to answer	39 (17.0)	428 (15.2)	2491 (15.4)	7997 (15.5)	14529 (15.9)	13986 (17.1)	4425 (19.1)
Missing	43 (18.8)	387 (13.7)	1615 (10.0)	3341 (6.5)	4562 (5.0)	3350 (4.1)	648 (2.8)
**Remoteness**							
Major cities	101 (44.1)	1314 (46.7)	7840 (48.4)	24712 (48.0)	41760 (45.8)	34901 (42.6)	8917 (38.5)
Inner regional	79 (34.5)	948 (33.7)	5280 (32.6)	17184 (33.4)	31766 (34.8)	30223 (36.9)	9092 (39.3)
Outer regional	40 (17.5)	477 (16.9)	2687 (16.6)	8485 (16.5)	15848 (17.4)	15261 (18.6)	4706 (20.3)
Remote/Very remote	8 (3.5)	75 (2.6)	389 (2.4)	1067 (2.1)	1800 (2.0)	1555 (1.9)	430 (1.9)
Missing	1 (0.4)	2 (0.1)	2 (0.01)	9 (0.02)	20 (0.02)	23 (0.03)	4 (0.02)
**Language spoken at home**							
English only	196 (85.6)	2430 (86.3)	14366 (88.7)	45980 (89.4)	82185 (90.1)	74659 (91.1)	21723 (93.8)
Language other than English	33 (14.4)	385 (13.7)	1832 (11.3)	5477 (10.6)	9009 (9.9)	7302 (8.9)	1426 (6.2)
Missing	0	1 (0.04)	0	0	0	2 (0.0)	0
**Private health insurance**							
Private (extras)	55 (24.0)	856 (30.4)	6481 (40.0)	23766 (46.2)	45220 (49.6)	42180 (51.5)	12340 (53.3)
Private (no extras)	13 (5.7)	241 (8.6)	1780 (11.0)	6818 (13.3)	13247 (14.5)	12492 (15.2)	3679 (15.9)
DVA health care	6 (2.6)	69 (2.4)	414 (2.6)	1141 (2.2)	1730 (1.9)	1180 (1.4)	271 (1.2)
Health care card	92 (40.2)	859 (30.5)	4019 (24.8)	10243 (19.9)	15684 (17.2)	13158 (16.1)	3650 (15.8)
None	63 (27.5)	791 (28.1)	3504 (21.6)	9489 (18.4)	15313 (16.8)	12953 (15.8)	3209 (13.8)
**History of chronic diseases** *							
No	145 (63.3)	1722 (61.2)	9615 (59.4)	31105 (60.5)	56666 (62.1)	51919 (63.3)	14457 (62.5)
Yes	84 (36.7)	1094 (38.8)	6583 (40.6)	20352 (39.5)	34528 (37.9)	30044 (36.7)	8692 (37.5)
**Prior PPH admission**							
No	219 (95.6)	2700 (95.9)	15468 (95.5)	49490 (96.2)	88569 (97.1)	79910 (97.5)	22628 (97.8)
Yes	10 (4.4)	116 (4.1)	730 (4.5)	1967 (3.8)	2625 (2.9)	2053 (2.5)	521 (2.2)

*Including melanoma, prostate/breast cancer and other cancers but not including non-melanoma skin cancer, heart disease, stroke, diabetes, asthma and osteoarthritis. Prior PPH was defined as any preventable hospitalisation which occurred 12 months prior to the study entry. Positive health behaviours were defined as current non-smoking, consuming less than 14 alcohol drinks per week, doing more than 2.5 hours of physical activity per week, consuming at least 5 servings of vegetables and 2 serving of fruit per day, having less than 8 hours of sitting per 24 hours and 7 hours or more of sleeping per 24 hours.

Because similar factors may influence both individuals' health behaviours and their disposition, capacity, and need to use health services and therefore risk of hospitalisation, a number of confounders were considered in the analysis [23]. These included sex, age (grouped into 5-year categories), educational level (did not complete high school, high school or equivalent, University or higher), marital status (single, married or partnered, windowed or separated), language spoken at home (English, language other than English), annual household income (<
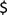
10,000, 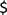
10,000–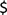
29,999, 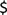
30,000–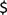
49,999, 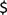
50,000–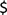
69,999, 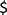
70,000 or more, and “I would rather not answer the question”) and their health insurance status (private health insurance with or without extras, Department of Veterans Affairs card, health care card, and none).

Current smoking status was based on responses to the questions: “Have you ever been a regular smoker?”, and (if yes) “Are you a regular smoker now?” Alcohol consumption was classified using responses to the question “How many alcoholic drinks do you have each week?” To adjust for under-reporting, alcohol consumption per week among drinkers was inflated by a factor of 9% [24]. Physical activity was assessed using the Active Australia Questionnaire [25] which elicits the number of hours and sessions of moderate and vigorous physical activity and walking per week. We weighted vigorous physical activity by a factor of two [25]. Information on fruit and vegetables intake was collected using the questions: “How many serves of vegetables/fruit or glasses of fruit juice do you usually eat/have each day?” Sitting and sleeping time were assessed using the questions: “How many hours in each 24-hour day do you usually spend sitting/sleeping (including at night and naps)?”

For each of the six health behaviours, we generated a binary exposure variable indicating “positive” health behaviour, according to national guidelines and the findings of previous studies: current non-smoking, consuming less than 14 alcoholic drinks per week (“low-to-moderate alcohol intake”) [26], doing more than 2.5 hours of intensity-weighted physical activity over at least 5 sessions per week (“sufficient physical activity”) [27], consuming at least 5 servings of vegetables and 2 servings of fruit per day (“sufficient fruit and vegetables intake”) [28], having less than 8 hours of sitting per day (“healthy sitting time”) [14] and 7 hours or more of sleeping per day (“healthy sleeping time”) [16].

We ascertained PPH admissions using linked hospital morbidity data, which captures all separations from public and private sector hospitals, based on the ICD10-AM diagnosis codes specified in the 2012 Australian National Healthcare Agreement PPH indicator [29] and categorised into chronic, acute and vaccine-preventable conditions (**Table S1**). All-cause mortality was ascertained from death registrations. Data were linked by the Centre for Health Record Linkage (http://www.cherel.org.au/) using probabilistic record linkage methods and commercially available software.

### Statistical analysis

Participants were followed from the date of recruitment to the date of first PPH admission or death, or December 2010 (the last date to which hospital data were available), whichever occurred first. Cox proportional hazards models with age as the underlying time variable [30] were used to estimate age- and sex- adjusted and multivariate adjusted hazard ratios (HRs) and 95% confidence intervals (CI) for PPH admission, overall and by category, according to individual positive health behaviours and the total number of these behaviours. One positive health behaviour was used as the reference category for the number of positive health behaviours because very few participants reported none of these behaviours. Other variables included in the fully adjusted models were: level of education, marital status, household income, remoteness, language other than English spoken at home, private health insurance, history of chronic diseases and PPH admission in the 12 months prior to study entry. Trend tests were assessed by fitting the number of positive health behaviours as a continuous term. We repeated similar analyses with all-cause mortality as the outcome in order to compare the magnitude of effects. To investigate the potential impacts of missing data and reverse causation, respectively, we ran models that included only participants with data available for all six health behaviours, and excluding participants who had PPH admissions or died in the first 12 months of follow-up. The point estimates were essentially unaltered (data not shown).

We calculated the population attributable risk (PAF) for all six positive health behaviours using the formula: 
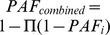
 [31] where PAF_i_ was calculated from the formula 
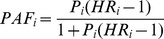
 [32] with HR_i_ was adjusted HR and P_i_ was national prevalence data for Australian adults: non-smoking: 84% [33]; low-to-moderate alcohol intake: 81% [33]; sufficient physical activity: 33% [33]; sufficient fruit and vegetables intake: 6% [33]; healthy sitting time: 67% [34]; healthy sleeping time: 36% [16]. 95% confidence intervals for the combined PAFs were derived using a substitution method [35]. Analyses were performed using SAS version 9.3.

### Ethical approval

Ethics approval for the APHID study was obtained from the NSW Population and Health Services Research Ethics Committee, Aboriginal Health and Medical Research Council of NSW Ethics Committee, and the University of Western Sydney Ethics Committee. The conduct of the 45 and Up Study was approved by the University of New South Wales Human Research Ethics Committee.

### Data sharing statement

The APHID study dataset has been constructed with the permission of each of the custodians of the respective source datasets and with specific ethical approval. The dataset could potentially be made available to other researchers if they obtain the necessary approvals. More information about these approvals is available from the authors on request.

## Results

Of the 267,031 participants in the 45 and Up Study in the dataset supplied for these analyses, 25 were excluded because they had inconsistent linked records, leaving 267,006 persons for analysis, with mean age at baseline 63 years (standard deviation 11 years).

Characteristics of participants according to number of health behaviours are shown in **Table 1**. Nearly 9% of participants reported undertaking all six of the positive health behaviours, while only 0.1% reported undertaking none of them. Women had on average more positive health behaviours than men (4.3 versus 3.9, P<0.001), and a greater proportion of women than men undertook all six positive health behaviours (73.5% versus 26.5%).

During an average of 2.7 years follow-up (interquartile range 2.3–2.9), 20971 (7.9%) participants had at least one PPH admission: 12971 (4.9%) for chronic, 8968 (3.4%) for acute and 585 (0.2%) for vaccine-preventable conditions. In the fully-adjusted model, non-smoking, sufficient physical activity, healthy sitting time and healthy sleeping time were all associated with reduced risk of PPH admission (**Table 2**). Low-to-moderate alcohol intake was associated with a higher risk of PPH admission. There was no significant association between sufficient fruit and vegetables intake and risk of PPH. Results were similar when stratified by gender, although in women the association between sufficient fruit and vegetables intake and lower risk of PPH admission attained borderline statistical significance (**Table 2**).

**Table 2:** Risk of potentially preventable hospitalisation (PPH) for individual health behaviours.

Health behaviour	Cohort N (% of total)	Any PPH admission		
		PPH admission (% of N)	Age and sex adjusted HR (95% CI)	Multivariable adjusted HR* (95% CI)
**All participants**				
Non-smoking	246267 (92.8)	19218 (7.8)	0.64 (0.61–0.68)	0.74 (0.69–0.78)
<14 alcohol drinks per week	212201 (81.2)	16958 (8.0)	1.23 (1.18–1.27)	1.12 (1.08–1.17)
≥2.5 hrs physical activity per week	179616 (67.3)	11247 (6.3)	0.64 (0.62–0.65)	0.74 (0.71–0.76)
≥2 servings of fruit and 5 servings of vegetables per day	59894 (23.5)	4511 (7.5)	0.96 (0.92–0.99)	0.99 (0.96–1.03)
<8 hrs sitting time per 24 hrs	184752 (74.1)	13645 (7.4)	0.82 (0.80–0.85)	0.86 (0.83–0.89)
≥7 hrs sleeping time per 24 hrs	220338 (84.3)	16522 (7.5)	0.85 (0.82–0.86)	0.94 (0.90–0.97)
**Men**				
Non-smoking	113679 (92.4)	10094 (8.9)	0.67 (0.63–0.72)	0.78 (0.72–0.85)
<14 alcohol drinks per week	87031 (71.5)	8172 (9.4)	1.21 (1.16–1.26)	1.11 (1.06–1.17)
≥2.5 hrs physical activity per week	82974 (67.0)	6080 (7.3)	0.63 (0.61–0.66)	0.73 (0.70–0.77)
≥2 servings of fruit and 5 servings of vegetables per day	18986 (16.2)	1794 (9.5)	1.02 (0.96–1.07)	1.01 (0.96–1.07)
<8 hrs sitting time per 24 hrs	83396 (71.5)	7161 (8.6)	0.84 (0.80–0.87)	0.86 (0.82–0.91)
≥7 hrs sleeping time per 24 hrs	103158 (85.0)	8893 (8.6)	0.82 (0.78–0.87)	0.91 (0.86–0.97)
**Women**				
Non-smoking	132588 (93.2)	9124 (6.9)	0.61 (0.57–0.66)	0.69 (0.63–0.75)
<14 alcohol drinks per week	125170 (89.7)	8786 (7.0)	1.29 (1.19–1.39)	1.15 (1.06–1.25)
≥2.5 hrs physical activity per week	96642 (67.5)	5167 (5.4)	0.63 (0.61–0.66)	0.74 (0.71–0.78)
≥2 servings of fruit and 5 servings of vegetables per day	40908 (29.7)	2717 (6.6)	0.92 (0.88–0.96)	0.98 (0.93–1.03)
<8 hrs sitting time per 24 hrs	101356 (76.5)	6484 (6.4)	0.81 (0.77–0.85)	0.86 (0.82–0.91)
≥7 hrs sleeping time per 24 hrs	117180 (83.6)	7629 (6.5)	0.88 (0.84–0.92)	0.95 (0.90–1.01)

PPH: potentially preventable hospitalisation.

*Adjusted for age, sex, education, marital status, income, remoteness, language other than English, private health insurance, history of chronic diseases, prior PPH admission and mutually adjusted for other health behaviours.

The risk of PPH admission during follow-up was significantly lower among participants reporting greater numbers of positive health behaviours: those reporting more than two positive health behaviours had between 16 and 46% lower risk of PPH admission compared with those reporting one health behaviour (**Table 3**). Results were similar when stratified by gender. Tests for trend showed a significant inverse linear association between number of positive health behaviours and risk of PPH admission, in all participants and in gender-specific analyses (**Table 3**).

**Table 3:** Risk of potentially preventable hospitalisation (PPH) by number of positive health behaviours.

Number of positive health behaviours	Cohort N (% of total)	Any PPH admission		
		PPH admission (% of N)	Age and sex adjusted HR (95% CI)	Multivariate adjusted HR* (95% CI)
**All participants**				
0	229 (0.1)	34 (14.8)	1.36 (0.96–1.93)	1.13 (0.80–1.61)
1	2816 (1.0)	365 (13.0)	1.00	1.00
2	16198 (6.1)	1822 (11.2)	0.82 (0.73–0.92	0.84 (0.75–0.94)
3	51457 (19.3)	5009 (9.7)	0.71 (0.64–0.79)	0.76 (0.69–0.85)
4	91194 (34.1)	7029 (7.7)	0.57 (0.51–0.63)	0.64 (0.58–0.72)
5	81963 (30.7)	5323 (6.5)	0.49 (0.44–0.54)	0.57 (0.51–0.63)
6	23149 (8.7)	1389 (6.0)	0.46 (0.41–0.52)	0.54 (0.48–0.61)
Test for trend			p<0.0001	p<0.0001
**Men**				
0	162 (0.1)	23 (14.2)	1.42 (0.92–2.19)	1.11 (0.72–1.71)
1	1672 (1.3)	200 (12.0)	1.00	1.00
2	9244 (7.5)	1049 (11.3)	0.87 (0.75–1.02)	0.94 (0.81–1.09)
3	27875 (22.5)	2793 (10.0)	0.74 (0.64–0.86)	0.83 (0.72–0.96)
4	45206 (36.5)	3837 (8.5)	0.60 (0.52–0.69)	0.70 (0.61–0.81)
5	33561 (27.1)	2629 (7.8)	0.52 (0.45–0.60)	0.63 (0.54–0.72)
6	6135 (5.0)	504 (8.2)	0.52 (0.44–0.61)	0.62 (0.52–0.73)
Test for trend			p<0.0001	p<0.0001
**Women**				
0	67 (0.1)	11 (16.4)	1.34 (0.73–2.47)	1.31 (0.71–2.41)
1	1144 (0.8)	165 (14.4)	1.00	1.00
2	6954 (4.9)	773 (11.1)	0.75 (0.63–0.89)	0.72 (0.61–0.85)
3	23582 (16.5)	2216 (9.4)	0.67 (0.60–0.78)	0.68 (0.58–0.80)
4	45988 (32.1)	3192 (6.9)	0.53 (0.45–0.62)	0.57 (0.49–0.67)
5	48402 (33.8)	2694 (5.6)	0.44 (0.38–0.52)	0.50 (0.42–0.58)
6	17014 (11.9)	885 (5.2)	0.41 (0.35–0.49)	0.47 (0.40–0.56)
Test for trend			p<0.0001	p<0.0001

Positive health behaviours were defined as current non-smoking, consuming less than 14 alcohol drinks per week, doing more than 2.5 hours of physical activity per week, consuming at least 5 servings of vegetables and 2 serving of fruit per day, having less than 8 hours of sitting per 24 hours and 7 hours or more of sleeping per 24 hours.

*Adjusted for age, sex, education, marital status, income, remoteness, language other than English, private health insurance, history of chronic diseases and prior PPH admission.

**Figure 1:**
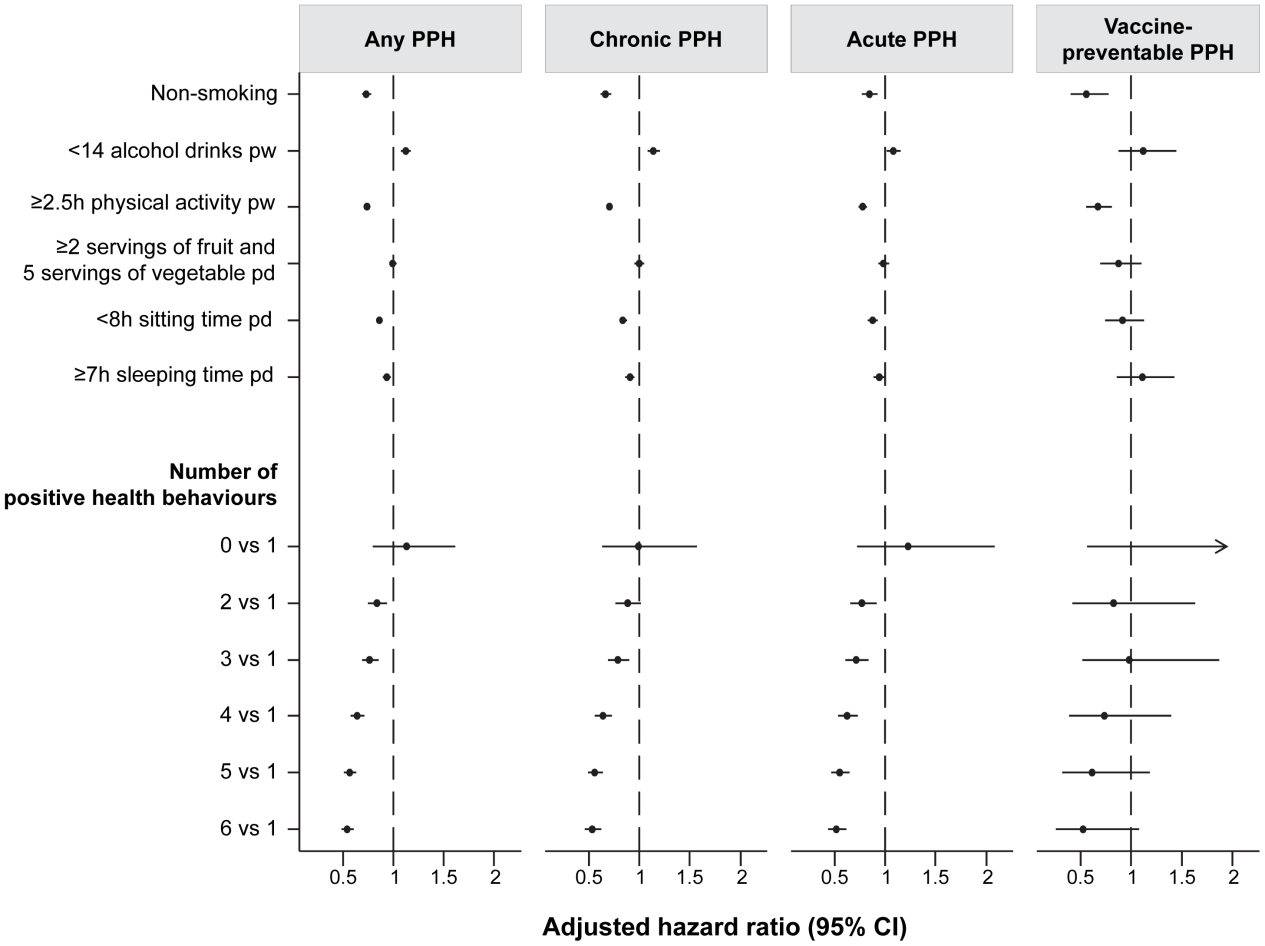
Risk of chronic, acute, and vaccine-preventable hospitalisations according to individual and combine of health behaviours. Hazard ratios (95% CI) estimated for the effect of each positive health behaviour on risk of PPH admission were adjusted for age, sex, education, marital status, income, remoteness, language other than English, private health insurance, history of chronic diseases, prior PPH admission and mutually adjusted for other health behaviours. Hazards ratios (95% CI) estimated for the effect of number of positive health behaviours on risk of PPH admission were adjusted for age, sex, education, marital status, income, remoteness, language other than English, private health insurance, history of chronic diseases and prior PPH admission.

Analyses stratified by category of PPH condition showed an inverse association between number of positive health behaviours and PPHs due to chronic, acute and vaccine-preventable conditions (P-trend all <0.001), although the estimates of PPHs due to vaccine-preventable conditions had wide confidence intervals (**Figure 1**).

Three per cent of participants died from any cause during follow up (N = 9133). Non-smoking, sufficient physical activity and healthy sitting time were individually associated with 27–44% lower risk of mortality in the fully adjusted model. No significant reduction was observed for low-to-moderate alcohol intake, sufficient fruit and vegetables intake or healthy sleeping time (**Table S2**). Mortality risk decreased linearly with increasing numbers of positive health behaviours. Those reporting more than two positive health behaviours had between 27–67% lower risk of death compared to those reporting one behaviour (**Table S3**). Results for mortality risk were similar when stratified by gender.

The estimated proportion of PPH admissions among Australian adults that were attributable to not undertaking the six positive health behaviours was 29% (95% CI 28%–31%) (35% (95% CI 34%–36%) of chronic PPH admissions, 25% (95% CI 24%–26%) of acute PPH admissions, and 37% (37%–38%) of vaccine-preventable PPH admissions). The comparable figure for all-cause mortality was 47% (95% CI 46%–48%).

## Discussion

In this large prospective study of people aged 45 years and over, we found that the risk of PPH admission decreased with increasing number of reported positive health behaviours (non-smoking, low-to-moderate alcohol consumption, sufficient physical activity, sufficient fruit and vegetables intake, healthy sitting time and healthy sleeping time). The magnitude of the association between these behaviours and PPH admission was similar for chronic, acute and vaccine-preventable PPH conditions. We estimated that approximately 29% of PPH admissions among Australian adults could be prevented if everyone undertook all six of these positive health behaviours. To our knowledge this is the first quantification of the relationship between individual and combined health behaviours and PPH admission.

Our findings with regard to mortality risk in relation to smoking status, physical activity and diet were congruent with those of previous longitudinal studies [10], [11], [13], [14], [16], and with previous studies in the 45 and Up Study population exploring sitting time [14] and sleeping time [16] The magnitude of the associations between similar sets of combined positive health behaviours and mortality risk in previous studies ranged from 42% [13] to 55% [36], consistent with our estimated 47% risk reduction for six compared with one positive behaviours.

However, there are few existing analyses of the association between health behaviours and PPH admission with which to compare with our results [7]. Previous studies have shown an association between obesity and higher risk of hospitalisation [37]–[39], both overall and for diabetes complications [40], and obesity is associated with sedentary lifestyle [41] and an unhealthy diet [42]. Other studies have clearly shown that smoking is associated with higher risk of hospitalisation for chronic obstructive pulmonary disease [43] and asthma [44]. Reduction in risk of hospitalisation for vaccine-preventable PPH conditions in association with positive health behaviours might at first appear to be counter-intuitive. However, many of these hospitalisations are for complications of influenza, which are more common in smokers and people with chronic comorbidities [45], [46].

Our results suggest that low-to-moderate alcohol consumption is associated with increased risk of PPH admissions, and this is not consistent with previous findings for mortality [10]–[12]. Measurement of alcohol consumption in older people is problematic [47] and our measure had some shortcomings. Participants were asked to report recent alcohol consumption, rather than lifetime consumption. Observational studies on recent alcohol consumption have found an inverse association with some conditions [48], [49]; whereas others measuring lifetime alcohol consumption have suggested a positive association [50]. One possible explanation for these discrepant findings could be that those participants with underlying medical conditions might have drunk more alcohol in their early life then reduced their intake in response to their disease diagnosis [48]. We inflated our measure of alcohol consumption by a factor of 9% to account for under-reporting [24], but it remains possible that measurement error in this population was more substantial than this [51].

Again, we had no previous estimates for the population proportion of PPH admissions that may be attributable to positive health behaviours with which to compare our findings. The estimated PAF for mortality in our study for the six health behaviours combined (47%) comparable to those previously reported for similar sets of health behaviours [11], [12], [36], [52], suggesting that PAF estimates generated from the 45 and Up Study are consistent with those from other cohorts internationally.

Strengths of our study included the longitudinal study design, the large sample size, the availability of data about a wide range of lifestyle factors and potential confounders, and comprehensive linkage to other health databases for ascertainment of outcomes [21]. The self-reported nature of the 45 and Up Study questionnaire introduces the potential for error in our measures of health behaviours, although the physical activity scale [25] and measure of sitting time [53] have previously been validated. However, the prospective design of the study and the independent ascertainment of outcomes through data linkage minimised the likelihood that such error was systematic and therefore introduced bias into our findings.

There was a relatively low participation rate in the 45 and Up Study (18%), raising a concern about the generalizability of our findings. A previous analysis that compared the 45 and Up Study cohort to a ‘representative’ population health survey found the subjects in both studies to be similar in terms of age, sex, remoteness of residence, country of birth, education, fruit consumption and obesity, but that participants in the 45 and Up Study had a lower prevalence of smoking, and higher rates of private health insurance, than the survey respondents. However, relative risk estimates relating to smoking and fruit consumption (among other variables) calculated using data from the two studies were very similar [54]. The large sample size in the 45 and Up Study provides substantial heterogeneity, and in these circumstances risk estimates calculated from internal comparisons within a cohort should remain valid [55].

We used a relatively short follow up period, and it is possible that people with illnesses likely to lead to PPH admission may have modified their health behaviours. Such reverse causation could result in a bias towards the null where individuals actively modify their behaviour in response to illness (e.g. by giving up smoking), but bias could operate in the opposite direction if illness itself influences the behaviour (e.g. by reducing capacity to undertake physical activity). However, we controlled for history of chronic disease and prior PPH in our models, and our results were unchanged when we excluded participants who had PPH admissions or died in the first 12 months of follow up.

The most important caveat with regard to our findings (and all previous studies exploring the relationship between health behaviours and mortality risk) is the potential for the observed associations to be influenced by residual confounding. We controlled for a wide range of confounding variables, but the potential remains for unmeasured “latent” variables such as health literacy, healthcare-seeking behaviour, compliance with health advice and risk-taking propensity to contribute to the observed associations. We would suggest that use of the number of positive health behaviours as an overall measure of “healthy behaviour” has advantages over approaches that use the individual component variables, because it does not make or invite assumptions about the independence or unique causal roles of specific health behaviours.

Our results provide novel evidence for the potential protective effect of positive health behaviours on PPH admission. They suggest that one of the key ways that primary care can contribute to reducing these admissions is through effective primary and secondary preventive interventions that modify individual behaviours and reduce risk. The results also indicate that effective population-level primary prevention strategies are likely to contribute to reducing the health system burden of PPH as these are currently conceptualised. Thus PPH should be viewed as a performance measure not just for primary care, but for the prevention system more broadly.

## Supporting Information

Table S1 Conditions included in the Australian National Healthcare Agreement potentially preventable hospitalisations performance indicator.
(DOCX)

Table S2 Risk of mortality for individual health behaviours.
(DOCX)

Table S3 Risk of mortality by number of positive health behaviours.
(DOCX)
