# Supplementary material for: Health Behaviours and Potentially Preventable Hospitalisation: A Prospective Study of Older Australian Adults
Source: PLoS One. 2014 Apr 1;9(4):e93111. doi: 10.1371/journal.pone.0093111 (PMC3972201; doi:10.1371/journal.pone.0093111)
Supplement: Table S1 — Conditions included in the Australian National Healthcare Agreement potentially preventable hospitalisations performance indicator. (DOCX) [file pone.0093111.s001.docx]

**Table S1: Conditions included in the Australian National Healthcare Agreement potentially preventable hospitalisations performance indicator**

| **PPH category** | **Condition** |
| --- | --- |
| **Chronic conditions** | Asthma |
|  | Congestive cardiac failure |
|  | Diabetes complications |
|  | Chronic obstructive pulmonary disease |
|  | Angina |
|  | Iron deficiency anaemia |
|  | Hypertension |
|  | Nutritional deficiencies |
|  | Rheumatic heart disease |
| **Acute conditions** | Dehydration and gastroenteritis |
|  | Pyelonephritis |
|  | Perforated/Bleeding ulcer |
|  | Cellulitis |
|  | Pelvic inflammatory diseases |
|  | Ear, nose and throat infection |
|  | Dental conditions |
|  | Appendicitis with generalised peritonitis |
|  | Convulsions and epilepsy |
|  | Gangrene |
| **Vaccine-preventable conditions** | Influenza and pneumonia |
|  | Other vaccine-preventable conditions |
