# Supplementary material for: Health Behaviours and Potentially Preventable Hospitalisation: A Prospective Study of Older Australian Adults
Source: PLoS One. 2014 Apr 1;9(4):e93111. doi: 10.1371/journal.pone.0093111 (PMC3972201; doi:10.1371/journal.pone.0093111)
Supplement: Table S2 — Risk of mortality for individual health behaviours. (DOCX) [file pone.0093111.s002.docx]

**Table S2: Risk of mortality for individual health behaviours**

| **Health behaviour** | **Cohort N**  **(% of total)** | **Death, n (%)** | **Mortality** | |
| --- | --- | --- | --- | --- |
|  |  |  | **Age and sex adjusted HR (95% CI)** | **Multivariable adjusted HR* (95% CI)** |
| **All participants** |  |  |  |  |
| Non-smoking | 246267 (92.8) | 8386 (3.4) | 0.46 (0.43-0.50) | 0.56 (0.51-0.61) |
| <14 alcohol drinks per week | 212201 (81.2) | 7200 (3.4) | 1.04 (0.98-1.10) | 0.95 (0.90-1.02) |
| ≥2.5 hrs physical activity per week | 179616 (67.3) | 3824 (2.1) | 0.50 (0.48-0.53) | 0.59 (0.56-0.62) |
| ≥2 servings of fruit and 5 servings of vegetables per day | 59894 (23.5) | 1693 (2.8) | 0.89 (0.84-0.94) | 0.95 (0.90-1.01) |
| <8 hrs sitting time per 24 hrs | 184752 (74.1) | 5260 (2.9) | 0.65 (0.62-0.68) | 0.73 (0.70-0.77) |
| ≥7 hrs sleeping time per 24 hrs | 220338 (84.3) | 7250 (3.3) | 0.96 (0.91-1.02) | 1.05 (0.98-1.12) |
| **Men** |  |  |  |  |
| Non-smoking | 113679 (92.4) | 5189 (4.6) | 0.46 (0.42-0.51) | 0.58 (0.52-0.66) |
| <14 alcohol drinks per week | 87031 (71.5) | 4211 (4.8) | 1.05 (0.99-1.12) | 0.97 (0.91-1.04) |
| ≥2.5 hrs physical activity per week | 82974 (67.0) | 2604 (3.1) | 0.53 (0.50-0.56) | 0.62 (0.59-0.66) |
| ≥2 servings of fruit and 5 servings of vegetables per day | 18986 (16.2) | 890 (4.7) | 0.98 (0.91-1.06) | 1.02 (0.94-1.10) |
| <8 hrs sitting time per 24 hrs | 83396 (74.5) | 3386 (4.1) | 0.69 (0.65-0.73) | 0.75 (0.70-0.80) |
| ≥7 hrs sleeping time per 24 hrs | 103158 (85.0) | 4572 (4.4) | 0.84 (0.78-0.91) | 0.94 (0.87-1.03) |
| **Women** |  |  |  |  |
| Non-smoking | 132588 (93.2) | 3197 (2.4) | 0.46 (0.40-0.53) | 0.52 (0.45-0.61) |
| <14 alcohol drinks per week | 125170 (89.7) | 2989 (2.4) | 1.00 (0.88-1.14) | 0.92 (0.80-1.06) |
| ≥2.5 hrs physical activity per week | 96642 (67.5) | 1220 (1.3) | 0.45 (0.42-0.49) | 0.53 (0.49-0.58) |
| ≥2 servings of fruit and 5 servings of vegetables per day | 40908 (29.7) | 803 (2.0) | 0.79 (0.73-0.86) | 0.89 (0.82-0.98) |
| <8 hrs sitting time per 24 hrs | 101356 (76.5) | 1874 (1.9) | 0.60 (0.55-0.65) | 0.71 (0.65-0.77) |
| ≥7 hrs sleeping time per 24 hrs | 117180 (83.6) | 2678 (2.3) | 1.15 (1.05-1.26) | 1.22 (1.10-1.36) |

*Adjusted for age, sex, education, marital status, income, remoteness, language other than English, private health insurance, history of chronic diseases, prior PPH admission and mutually adjusted for other health behaviours.
