# Supplementary material for: Health Behaviours and Potentially Preventable Hospitalisation: A Prospective Study of Older Australian Adults
Source: PLoS One. 2014 Apr 1;9(4):e93111. doi: 10.1371/journal.pone.0093111 (PMC3972201; doi:10.1371/journal.pone.0093111)
Supplement: Table S3 — Risk of mortality by number of positive health behaviours. (DOCX) [file pone.0093111.s003.docx]

**Table S3: Risk of mortality by number of positive health behaviours.**

| **Number of positive health behaviours** | **Cohort N**  **(% of total)** | **Mortality** | | |
| --- | --- | --- | --- | --- |
|  |  | **Deaths, n (%)** | **Age and sex adjusted HR (95% CI)** | **Multivariate adjusted HR* (95% CI)** |
| **All participants** |  |  |  |  |
| 0 | 229 (0.1) | 15 (6.5) | 0.97 (0.58-1.64) | 0.83 (0.49-1.39) |
| 1 | 2816 (1.0) | 227 (8.1) | 1.00 | 1.00 |
| 2 | 16198 (6.1) | 1029 (6.4) | 0.71 (0.61-0.82) | 0.73 (0.63-0.84) |
| 3 | 51457 (19.3) | 2634 (5.1) | 0.59 (0.51-0.67) | 0.64 (0.56-0.73) |
| 4 | 91194 (34.1) | 3106 (3.4) | 0.43 (0.38-0.50) | 0.50 (0.43-0.57) |
| 5 | 81963 (30.7) | 1730 (2.1) | 0.30 (0.26-0.35) | 0.36 (0.31-0.41) |
| 6 | 23149 (8.7) | 392 (1.7) | 0.28 (0.24-0.33) | 0.33 (0.28-0.39) |
| Test for trend |  |  | <0.0001 | <0.0001 |
| **Men** |  |  |  |  |
| 0 | 162 (0.1) | 10 (6.2) | 0.86 (0.46-1.64) | 0.71 (0.38-1.35) |
| 1 | 1672 (1.4) | 157 (9.4) | 1.00 | 1.00 |
| 2 | 9244 (7.5) | 652 (7.1) | 0.66 (0.55-0.78) | 0.71 (0.60-0.85) |
| 3 | 27875 (22.5) | 1604 (5.8) | 0.51 (0.43-0.60) | 0.59 (0.50-0.70) |
| 4 | 45206 (36.5) | 1974 (4.4) | 0.39 (0.33-0.45) | 0.47 (0.40-0.55) |
| 5 | 33561 (27.1) | 1093 (3.3) | 0.28 (0.24-0.33) | 0.35 (0.30-0.42) |
| 6 | 6135 (4.9) | 203 (3.3) | 0.27 (0.22-0.34) | 0.35 (0.28-0.43) |
| Test for trend |  |  | <0.0001 | <0.0001 |
| **Women** |  |  |  |  |
| 0 | 67 (0.1) | 5 (7.5) | 1.19 (0.48-2.96) | 1.09 (0.44-2.70) |
| 1 | 1144 (0.8) | 70 (6.1) | 1.00 | 1.00 |
| 2 | 6954 (4.9) | 377 (5.4) | 0.81 (0.63-1.04) | 0.77 (0.59-0.99) |
| 3 | 23582 (16.5) | 1030 (4.4) | 0.74 (0.58-0.95) | 0.72 (0.57-0.93) |
| 4 | 45988 (32.1) | 1132 (2.5) | 0.53 (0.42-0.68) | 0.55 (0.43-0.70) |
| 5 | 48402 (33.8) | 637 (1.3) | 0.34 (0.26-0.43) | 0.36 (0.28-0.47) |
| 6 | 17014 (11.9) | 189 (1.1) | 0.30 (0.23-0.40) | 0.33 (0.25-0.44) |
| Test for trend |  |  | <0.0001 | <0.0001 |

Positive health behaviours were defined as current non-smoking, consuming less than 14 alcohol drinks per week, doing more than 2.5 hours of physical activity per week, consuming at least 5 servings of vegetables and 2 serving of fruit per day, having less than 8 hours of sitting per 24 hours and 7 hours or more of sleeping per 24 hours. *Adjusted for age, sex, education, marital status, income, remoteness, language other than English, private health insurance, history of chronic diseases and prior PPH admission.
